# The effectiveness and safety of low-level laser therapy on breast cancer–related lymphedema: An overview and update of systematic reviews

**DOI:** 10.1007/s10103-021-03446-3

**Published:** 2021-11-15

**Authors:** Yuping Wang, Yonggui Ge, Wenting Xing, Junping Liu, Jiqi Wu, Haijuan Lin, Yaqin Lu

**Affiliations:** Department of Rehabilitation, The First Hospital of Lanzhou University, No. 1 Donggang West Road, Chengguan District, Lanzhou, 730000 People’s Republic of China

**Keywords:** Low-level laser therapy, Photobiomodulation therapy, Breast cancer–related lymphedema, Overview, Systematic reviews

## Abstract

The objective of our overview of systematic reviews was to critically analyze the evidence from existing systematic reviews investigating the effectiveness and safety of low-level laser therapy (LLLT) in patients with breast cancer–related lymphedema (BCRL). In addition, an updated and comprehensive systematic review was conducted, which aimed to provide updated evidence about this topic. PubMed, EMBASE, and Cochrane Library databases were systematically searched for systematic reviews and randomized controlled trials (RCTs) investigating the effectiveness and safety of LLLT in patients with BCRL. The methodological quality for each of included systematic reviews or RCTs was assessed using the Assessing the Methodological Quality of Systematic Reviews 2 (AMSTAR 2) tool or Cochrane risk of bias tool, respectively. The updated systematic review separately compared the effectiveness of LLLT to each of active or negative interventions. Data were pooled with random-effects models for each outcome per comparison. The evidence quality of outcomes was assessed using the Grading of Recommendations, Assessment, Development and Evaluation (GRADE) or GRADE-Confidence in the Evidence from Reviews of Qualitative research (GRADE-CERQual) for quantitative studies and qualitative studies, respectively. Seven systematic reviews and ten RCTs met the eligibility criteria. Conflicting results regarding the effectiveness of LLLT were presented by the overview of systematic reviews. The AMSTAR 2 showed that the methodological quality of included systematic reviews was low or critically low quality due to one or more critical weaknesses. The GRADE and GRADE-CERQual showed that the evidence quality was low to very low for most outcomes. The updated systematic review showed that LLLT may offer additional benefits as compared to compression therapies (pneumatic compression or compression bandage), placebo laser, or no treatment for patients with BCRL. However, when compared to other types of active interventions, LLLT did not improve outcomes significantly. None of the treatment-related adverse event was reported. Many trials had a high or unclear risk of bias for two or more items, and our updated systematic review showed low quality of evidence per outcome using GRADE approach. Due to insufficient data and poor quality of evidence, there is uncertain to reach these conclusions that LLLT is superior to another active or negative intervention and is safe. More RCTs of high methodological quality, with large sample sizes and long-term follow-up, are needed to inform clinical guidelines and routine practice.

**Supplementary Information:**

The online version contains supplementary material available at 10.1007/s10103-021-03446-3.

## Introduction

Breast cancer is the most common malignancy and the leading cause of cancer-related mortality among women worldwide [1]. Despite advancements in treatments for breast cancer have decreased the risk of metastasis and improved survival in women, a considerable number of breast cancer survivors are forced to endure lifelong problems, such as lymphedema. Breast cancer–related lymphedema (BCRL) is a much-feared sequela characterized by chronic accumulation of protein-rich fluid in the interstitial spaces arising from impairment of the lymphatic system [2]. DiSipio et al. [3] reported an overall BCRL incidence rate of 16.6% (95% CI: 13.6 to 20.2) in individuals 3 months to 20 years after diagnosis. Despite modern surgical techniques (such as sentinel node biopsy [4] and axillary reverse mapping[5]) are effective at reducing the incidence of BCRL, BCRL remains a major problem. Although the most visible manifestation of BCRL is swelling, survivors often experience multiple symptoms, including pain, heaviness, tightness, numbness, stiffness, and fatigue in the affected limb [6, 7]. Consequences of these symptoms result in poor psychological health[8], diminished physical function [9], and decreased quality of life [10].

Currently, there is a variety of non-invasive treatment strategies, of which complex decongestive therapy (CDT) is nowadays regarded as the standard therapy for patients with BCRL [11]. CDT is individualized for each patient, but it typically includes manual lymphatic drainage (MLD), compression bandaging, exercise, skin care, and patient education. Nevertheless, it is also considered time-consuming and requires a high level of compliance. Therefore, an effective and convenient therapeutic regime for better management of BCRL is required. In the last 20 years, low-level laser therapy (LLLT), as known as photobiomodulation therapy (PBMT), has become increasingly popular in the supportive care of patients with breast cancer or BCRL [12]. LLLT is a non-invasive, painless, and can be easily administered therapy that utilizes wavelengths of red or near infrared light between 650 and 1000 nm to deliver low irradiance and doses to the target tissue. LLLT is believed to stimulate lymphatic motricity, promote lymphangiogenesis, and prevent tissue fibrosis [13–15], which facilitate removal of excess protein-rich fluid. LLLT is also speculated to stimulate macrophage cells and immune system [16, 17] which decrease the risk of infection.

In November 2006, the Food and Drug Administration (FDA) approved the use of the LLLT for treatment of post-mastectomy lymphedema. However, there have been contradictory findings from systematic reviews examining the effect of LLLT on lymphedema outcomes. The objective of our work consists of two stages. First, we conducted an overview of systematic reviews to critically analyze the evidence from existing systematic reviews concerning the effectiveness and safety of LLLT in patients with BCRL. The second stage involved updating a well-designed and comprehensive systematic review about this topic.

## Methods

This study was conducted according to the Preferred Reporting Items for Systematic Reviews and Meta-Analyses (PRISMA)[18, 19] statements and the recommendations of the Cochrane Collaboration Handbook [20].

### Eligibility criteria

#### Types of studies

Systematic reviews, which had to be clearly identified by the authors as a “systematic review” or “meta-analysis” in either the title or abstract of the review and conduct the assessment of risk of bias, were included for an overview of systematic reviews. Randomized controlled trials (RCTs) were included for an updated systematic review.

#### Types of participants

All study participants had to have a definite diagnosis of BCRL subsequent to any type of surgery, radiotherapy, or combination of these. We had no restrictions on age and gender.

#### Types of interventions

Intervention group was LLLT as a single therapy or combined therapy was included. There was no restriction regarding the control group, including no treatment or waiting list, placebo/sham laser therapy, and conventional therapies.

#### Types of outcome measures

The included systematic reviews or RCTs had to focused on the effectiveness of LLLT on limb circumference/volume, fluid distribution, tissue resistance, lymphedema-related subjective symptoms, physical function (grip strength, shoulder mobility), activity disability and quality of life, or adverse events.

### Search strategy

A comprehensive computer-aided literature search was undertaken in PubMed, Embase, and Cochrane library databases for systematic reviews (from inception to 25 January 2021) and RCTs (from inception to 15 March 2021), without restrictions regarding publication status or language. The search combined the following search terms: “breast neoplasms,” “lymphedema,” “breast cancer lymphedema,” “low-level light therapy,” “laser therapy,” “lasers,” “meta-analysis,” “systematic review,” and multiple synonyms for each term with slight modifications for individual searches in each database. Additional articles were sought by manual screening of reference lists of relevant literatures. Professionals working in the field were contacted to identify potential articles. The search strategy for the PubMed database is presented in Supplemental Table 1.

### Study selection

Two authors (W.J.Q and L.H.J) independently determined the eligibility of each study. Two authors first screened the titles and abstracts of citation. Then two authors reviewed the full-text articles for each citation and assessed against the eligibility criteria. In case of discrepancies, consensus was achieved by discussion. If consensus could not be reached, a third author (L.Y.Q) was consulted.

### Data extraction

For each included review, two authors (G.Y.G and X.W.T) independently extracted the data on the details: the first author, publication year, country, number of included trials and participants, treatment interventions, control interventions, outcomes, quality assessment tools, main results, and adverse events. For each included RCT, we extracted the first author, publication year, inclusion criteria, number of patients, intervention group, control group, outcomes reported, and assessment times. We also extracted mean change difference of outcome data between baseline and post-treatment or follow-up periods when compared intervention group with control group. Differences between the review authors were settled by discussion, and a third author (L.Y.Q) was consulted if differences persisted. Where required, we contacted study authors for additional information.

### Assessment of methodological quality

Two authors (W.Y.P and G.Y.G) independently assessed the methodological quality of each of included systematic reviews using the Assessing the Methodological Quality of Systematic Reviews 2 (AMSTAR 2) tool [21]. The overall methodological quality of included systematic reviews was classified as high, moderate, low, or critically low. Two other authors (X.W.T and L.J.P) independently appraised the methodological quality of each of the included primary trials using Cochrane risk of bias tool [22]. Every domain was classified as high, low, and unclear risk of bias. Disagreements regarding by-item and overall rating of quality were resolved by consensus or a third reviewer adjudication (L.Y.Q).

### Assessment of the evidence quality

Four authors (W.Y.P, G.Y.G, X.W.T and L.J.P) independently assessed the strength of evidence associated with outcomes using two different approaches. The Grading of Recommendations, Assessment, Development and Evaluation (GRADE) [23] was utilized for included quantitative reviews and our updated systematic review, whereas the GRADE-Confidence in the Evidence from Reviews of Qualitative research (GRADE-CERQual) [24] was utilized for included qualitative reviews. For each outcome, the evidence can be graded as high, moderate, low, or very low. Discrepancies between investigators were resolved by discussion or by a third reviewer (L.Y.Q) in cases when a consensus was not reached.

### Statistical analysis

Interventions varied substantially between studies, and we classified them into seven broad categories for separately comparing the LLLT to each of active or negative interventions. We undertook a quantitative evaluation of data with random-effects model using the Review Manager Software version 5.3. We expressed dichotomous data for each arm in a particular study as a proportion and the treatment effect as an odd ratio (*OR*) with 95% confidence intervals (*CI*), calculated using Mantel–Haenszel methods. We expressed continuous data for each arm in a particular study as a mean and standard deviation and the treatment effect as the standardized mean difference (*SMD*). Finally, we created a bubble plot to present evidence base using Microsoft office Excel 2016 software (Microsoft Corp., Redmond, WA, www.microsoft.com).

## Results

### Overview of systematic reviews

#### Search results

**Fig. 1:**
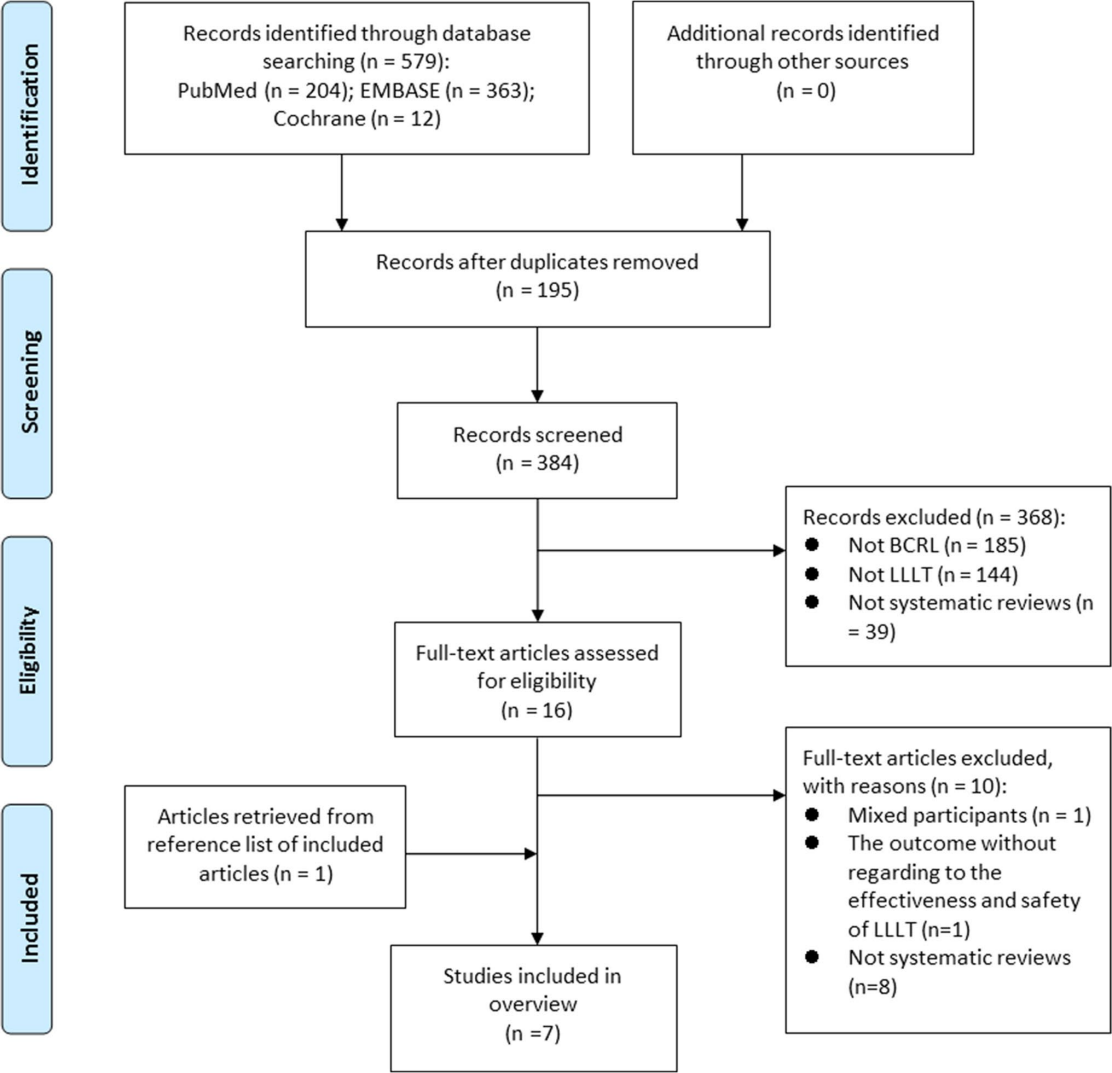
PRISMA flow diagram illustrating the selection of systematic reviews. *BCRL* breast cancer–related lymphedema, *LLLT* low-level laser therapy

Overall, 579 records were retrieved from the three electronic databases. After removing duplicates and screening titles and abstracts, 16 publications were identified as potentially eligible. Full-texts were retrieved for further assessment. According to the inclusion criteria, seven reviews were included in this overview (the reasons for exclusion in Supplemental Table 2). The PRISMA flow diagram of selected systematic reviews was illustrated in Fig. 1.

#### Study characteristics

The characteristics of seven reviews included in this overview were presented in Table 1. All reviews were published in English between 2007 and 2019 and undertaken in six different areas including China [25], New Zealand [26], America [27], Brazil [28, 29], Egypt [30], and Britain [31], respectively. Two reviews employed meta-analysis methods as data synthesis, yet the remaining five that did not use it were narrative systematic reviews. The number of primary trials for LLLT included in each review ranged from 3 to 11, and the sample sizes ranged from 71 to 359. Three reviews [25, 28, 29] included only RCTs, and four [26, 27, 30, 31] included both RCTs and observational studies.

**Table 1:** Main characteristics of included systematic reviews

Author (Year)	Country	Trials (Sample size)	Intervention		Outcomes	Quality assessment tool	Main results	Adverse effects
			Treatment group	Control group				
Chen (2019)	China	9 RCTs (316 participants)	LLLT; LLLT + conventional therapy	Conventional therapies: pneumatic compression, limb exercise, manual lymphatic drainage; Placebo laser therapy; No treatment;	Primary outcome: the difference in the limb circumference or volume Secondary outcomes: grip strength and pain scores	RoB 2.0	LLLT demonstrated a slight improvement in reducing arm circumference and arm volume However, there was no significant difference in the reduction of the limb circumference, limb volume, grip strength or pain scores after treatment, and follow-up between the LLLT and control groups	
Baxter (2017)	New Zealand	7 RCTs + 4 observational studies (359 participants)	LLLT	Conventional therapies: compression bandages, pneumatic compression, manual lymphatic drainage Placebo laser therapy No treatment	Primary outcome: limb circumference, limb volume Secondary outcomes: pain intensity and range of motion	PEDro scale	LLLT in the management of BCRL is more effective for limb edema reduction than sham laser therapy and no treatment at a short-term follow-up LLLT were not more effective than other conventional treatments	
Smoot (2015)	American	7 RCTs + 2 observational studies (289 participants)	LLLT	Conventional therapies: intermittent compression, compression garment, manual lymphatic drainage Placebo laser therapy No treatment	Reduction in limb volume, pain reduction	PEDro scale	There was greater reduction in limb volume with treatment including LLLT versus not including LLLT There was no statistically significant difference in amount of pain reduction after LLLT as compared to other treatments	Six of the nine studies reported on adverse events or discussed harm. The total incidence of cellulitis reported in the included studies was two per group
Monteiro (2014)	Brazil	5 RCTs (189 participants)	LLLT	Conventional therapies: manual lymphatic drainage Placebo laser therapy	Limb circumference, limb volume, extracellular fluid, subjective symptoms: pain, heaviness, psychological and physical symptoms, quality of life	PEDro scale	All the articles included in this review resulted in reduced circumference or volume of the affected limb after LLLT However, results regarding pain were not consistent	
E Lima (2014)	Brazil	4 RCTs (149 participants)	LLLT	Conventional therapies: pneumatic compression Placebo laser therapy No treatment	Limb volume; tissue hardness; range of motion; grip strength; subjective symptoms: pain, heaviness, DASH questionnaire symptoms	PEDro scale	In all studies, LLLT showed favorable results in limb volume reduction as compared with the control group. Also, significant decrease in tissue hardness was observed in two studies LLLT failed to show improvement of subjective symptoms in all but one study	No adverse reactions were reported
Omar (2012)	Egypt	5RCTs + 3 observational studies (220 participants)	LLLT	Conventional therapies: compression therapy Placebo laser therapy No treatment	Limb circumference; limb volume; fluid distribution; tissue resistance; shoulder mobility; grip strength; subjective symptoms	PEDro scale	Five studies with moderate to strong evidences demonstrated the effectiveness of LLLT for the management of BCRL. A dose of 1–2 J/cm^2^ per point applied to several points covering the fibrotic area can reduce limb volume following BCRL	
Moseley (2007)	Britain	1RCT + 2 observational studies (71 participants)	LLLT	Placebo laser therapy No control group	Limb volume; subjective symptoms; daily living activities	PEDro scale, NOS	These three studies demonstrate that benefits including volume reduction, improved subjective symptoms and quality of life can be derived from either concentrated or scanning laser therapy	

*RCTs*, randomized controlled trials; *LLLT*, low-level laser therapy; *RoB*, risk of bias; *PEDro*, physiotherapy evidence databases; *BCRL*, breast cancer–related lymphedema; *NOS*, Newcastle–Ottawa Scale

The intervention groups were mostly LLLT and conventional therapy in the treatment group, and the control groups were mostly conventional therapy (compression therapy, manual lymphatic drainage, limb exercise), placebo laser therapy, and no treatment. The methodological quality assessment scales varied across the included reviews: five used the Physiotherapy Evidence Database (PEDro) scale [26–31], and one adopted the Risk of Bias (RoB) 2.0 scale [25], and one used both the PEDro scale and Newcastle–Ottawa Scale (NOS) [31].

The primary studies overlap of included reviews was reported in Table 2. One [32] of the primary studies overlapped across seven of the included reviews; two [33, 34] overlapped across six reviews; two [35, 36] overlapped across five reviews; two [37, 38] overlapped across four reviews; and three [39–41] overlapped across three reviews; three [42–44] did not overlap.

**Table 2:** Primary trials for LLLT overlap in systematic reviews

Primary study	Systematic review with meta-analysis		Systematic review				
	Chen (2019)	Smoot (2015)	Baxter (2017)	Monteiro (2014)	E Lima (2014)	Omar (2012)	Moseley (2007)
RCTs							
Baxter (2018)	√						
Storz (2017)	√						
Rinder (2013)	√	√	√	√			
Omar (2011)	√	√	√	√		√	
Kozanoglu (2009)	√	√	√		√	√	
Lau (2009)	√	√	√	√	√	√	
Maiya (2008)	√	√	√				
Kaviani (2006)	√	√	√	√	√	√	
Carati (2003)	√	√	√	√	√	√	√
Observational studies							
Mayrovitz (2011)			√				
Dirican (2011)		√	√			√	
Piller (1995)			√			√	√
Piller (1998)		√	√			√	√

*LLLT*, low-level laser therapy; *RCTs*, randomized controlled trials

#### Methodological assessment

**Fig. 2:**
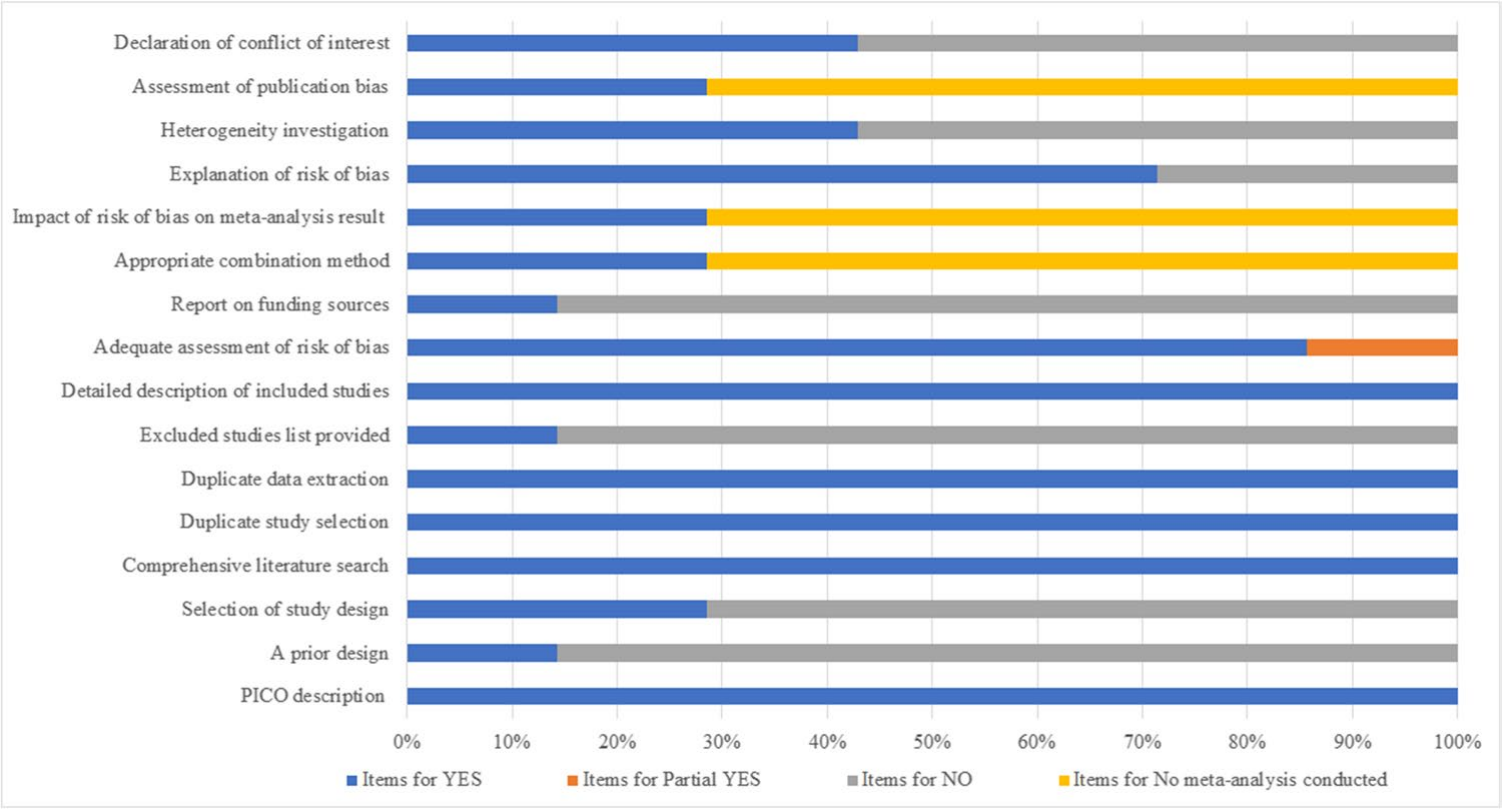
Methodological quality of the included systematic reviews with AMSTAR 2 checklist

The AMSTAR 2 score of including systematic reviews is presented in Table 3 and Fig. 2. According to the evaluation criteria, two (28.6%) [25, 26] reviews were of low quality and five (71.4%) [27–31] of critically low quality. All the reviews explicitly described the components of PICO, used comprehensive search strategies, conducted the study selection and data extraction in duplicate, and describe the included studies in adequate detail. However, only one [25] (14.3%) review had an explicit statement regarding review methods prior to the conduct of the review, one [26] (14.3%) listed the excluded studies and provided the funding information of the included studies, two [25, 30] (28.6%) explained the reasons for the study design selection, three [25–27] (42.9%) explained or discussed any heterogeneity, and three [25, 26, 30] (42.9%) declared the conflicts of interest.

**Table 3:** Result of the AMSTAR 2 assessments

Study	AMSTAR 2 domains																Overall quality
	1	2	3	4	5	6	7	8	9	10	11	12	13	14	15	16	
Chen (2019)	Y	Y	Y	Y	Y	Y	N	Y	Y	N	Y	Y	Y	Y	Y	Y	L
Baxter (2017)	Y	N	N	Y	Y	Y	Y	Y	PY	Y	NMA	NMA	Y	Y	NMA	Y	L
Smoot (2015)	Y	N	N	Y	Y	Y	N	Y	Y	N	Y	Y	Y	Y	Y	N	CL
Monteiro (2014)	Y	N	N	Y	Y	Y	N	Y	Y	N	NMA	NMA	N	N	NMA	N	CL
E Lima (2014)	Y	N	N	Y	Y	Y	N	Y	Y	N	NMA	NMA	Y	N	NMA	N	CL
Omar (2012)	Y	N	Y	Y	Y	Y	N	Y	Y	N	NMA	NMA	Y	N	NMA	Y	CL
Moseley (2007)	Y	N	N	Y	Y	Y	N	Y	Y	N	NMA	NMA	N	N	NMA	N	CL

**Domains**: 1 = Did the research questions and inclusion criteria for the review include the components of PICO?

2 = Did the report of the review contain an explicit statement that the review methods were established prior to the conduct of the review and did the report justify any significant deviations from the protocol?

3 = Did the review authors explain their selection of the study designs for inclusion in the review?

4 = Did the review authors use a comprehensive literature search strategy?

5 = Did the review authors perform study selection in duplicate?

6 = Did the review authors perform data extraction in duplicate?

7 = Did the review authors provide a list of excluded studies and justify the exclusions?

8 = Did the review authors describe the included studies in adequate detail?

9 = Did the review authors use a satisfactory technique for assessing the risk of bias (RoB) in individual studies that were included in the review?

10 = Did the review authors report on the sources of funding for the studies included in the review?

11 = If meta-analysis was performed, did the review authors use appropriate methods for statistical combination of results?

12 = If meta-analysis was performed, did the review authors assess the potential impact of RoB in individual studies on the results of the meta-analysis or other evidence synthesis?

13 = Did the review authors account for RoB in individual studies when interpreting/discussing the results of the review?

14 = Did the review authors provide a satisfactory explanation for, and discussion of, any heterogeneity observed in the results of the review?

15 = If they performed quantitative synthesis did the review authors carry out an adequate investigation of publication bias (small study bias) and discuss its likely impact on the results of the review?

16 = Did the review authors report any potential sources of conflict of interest, including any funding they received for conducting the review?

**Answers:** *Y* = Yes; *PY* = Partial Yes; *N* = No; *NMA* = No meta-analysis conducted; *CL* critically low; *L* low; *H* high

#### Evidence quality of outcomes

The quality of the evidence reported from two quantitative reviews, assessed using the GRADE method, is summarized in Table 4. Based on the different control groups or assessment times, the quality of the evidence for the outcomes varied widely. The GRADE assessment revealed that all outcomes provided low- to very low-quality evidence. The reasons for downgrade were as follow: (1) For 15 (15/15, 100.0%) outcomes, risk of bias (incomplete reporting of randomization, no blinding and allocation concealment, and failure to adhere to the intention-to-treat) decreased the validity of the GRADE approach. (2) For five (5/15, 33.3%) outcomes, we downgraded the quality of evidence based on inconsistency owing to unexplained high heterogeneity. (3) For 15 (15/15, 100%) outcomes, we downgraded the quality of evidence based on imprecision owing to the wide confidence intervals or optimal information size criterion not met (< 300).

**Table 4:** Result of the GRADE assessments in included systematic reviews

Outcomes	Study	Intervention	Control	Assessment times	Effect (95% CI)	Studies (participants)	Risk of bias	Inconsistency	Indirectness	Imprecision	Publication bias	GRADE quality of evidence
Limb circumference	Chen (2019)	LLLT; LLLT + conventional therapy	Conventional therapies Placebo laser therapy	Post-treatment	*SMD* − 0.47 (− 1.34, 0.39)	3 (117)	− 1^(1c,1e)^	− 1^(2)^	0	− 1^(4b)^	0	Very low quality
	Chen (2019)	LLLT; LLLT + conventional therapy	Conventional therapies Placebo laser therapy	1-month	*SMD* − 0.52 (− 0.52, 0.42)	2 (67)	− 1^(1b,1c,1e)^	− 1^(2)^	0	− 1^(4b)^	0	Very low quality
	Chen (2019)	LLLT	Conventional therapies	3-month	*SMD* − 0.33 (− 0.88, 0.23)	1 (50)	− 1^(1a,1c,1e)^	0	0	− 1^(4b)^	0	Low quality
Limb volume	Chen (2019)	LLLT	Placebo laser therapy; no treatment	Post-treatment	*SMD* 0.04 (− 0.32, 0.41)	3 (122)	− 1^(1b,1c)^	0	0	− 1^(4b)^	0	Low quality
	Smoot (2015)	LLLT	Conventional therapies Placebo laser therapy; no treatment	Post-treatment	*SMD* 0.62 (− 0.97, − 0.28)	4 (138)	− 1^(1b,1c,1e)^	0	0	− 1^(4b)^	0	Low quality
	Chen (2019)	LLLT	Placebo laser therapy; no treatment	1-month	*SMD* − 0.53 (− 1.10, 0.04)	2 (82)	− 1^(1b,1c)^	0	0	− 1^(4b)^	0	Low quality
Grip strength	Chen (2019)	LLLT	Conventional therapies; Placebo laser therapy;	Post-treatment	*MD* 1.18 (− 2.16, 4.52)	3(140)	− 1^(1b,1c,1e)^	− 1^(2)^	0	− 1^(4a,4b)^	0	Very low quality
	Chen (2019)	LLLT	Placebo laser therapy;	1-month	*MD* − 1.93 (− 5.15, 1.29)	1(40)	− 1^(1b,1e)^	0	0	− 1^(4a,4b)^	0	Low quality
	Chen (2019)	LLLT	Placebo laser therapy;	2-month	*MD* 0.67 (− 2.72, 4.06)	1(40)	− 1^(1b,1e)^	0	0	− 1^(4a,4b)^	0	Low quality
	Chen (2019)	LLLT	Conventional therapies; Placebo laser therapy	3-month	*MD* − 0.89 (− 3.04, 1.25)	2(90)	− 1^(1a,1c,1e)^	0	0	− 1^(4a,4b)^	0	Low quality
Pain	Chen (2019)	LLLT; LLLT + conventional therapy	Conventional therapies Placebo laser therapy	Post-treatment	*MD* − 0.14 (− 0.58, 0.31)	4(168)	-1^(1b,1c,1e)^	0	0	− 1^(4b)^	0	Low quality
	Smoot (2015)	LLLT	Conventional therapies	Post-treatment	*SMD* − 1.21 (− 4.51, 2.10)	2(67)	− 1^(1a,1c,1e)^	− 1^(2)^	0	− 1^(4a,4b)^	0	Very low quality
	Chen (2019)	LLLT; LLLT + conventional therapy	Conventional therapies; Placebo laser therapy	1-month	*MD* 0.21 (− 0.27, 0.68)	3(118)	− 1^(1b)^	0	0	− 1^(4b)^	0	Low quality
	Chen (2019)	LLLT	Placebo laser therapy	2-month	MD 0.00 (− 1.87, 1.87)	1(40)	− 1^(1b,1e)^	0	0	− 1^(4a,4b)^	0	Low quality
	Chen (2019)	LLLT	Conventional therapies Placebo laser therapy	3-month	MD 0.01 (− 0.99, 1.02)	3(151)	− 1^(1b,1e)^	− 1^(2)^	0	− 1^(4b)^	0	Very low quality

*GRADE*, Grading of Recommendations, Assessment, Development and Evaluation; *CI*, confidence interval; *LLLT*, low-level laser therapy; *SMD*, standardized mean difference; *MD*, mean difference

High quality: further research is very unlikely to change our confidence in the estimate of effect

Moderate quality: further research is likely to have an important impact on our confidence in the estimate of effect and may change the estimate

Low quality: further research is very likely to have an important impact on our confidence in the estimate of effect and is likely to change the estimate

Very low quality: any estimate of effect is very uncertain

Risk of bias: (1a) incomplete reporting of random sequence generation; (1b) no allocation concealment; (1c) no blinding for subjects, therapists or assessors; (1d) loss to follow-up; (1e) failure to adhere to the intention-to-treat; (1f) selective reporting

Inconsistency: (2) unexplained high heterogeneity of results

Indirectness: (3a) differences in therapeutic methods between intervention and control groups; (3b) surrogate outcome

Imprecision: (4a) wide confidence intervals; (4b) optimal information size criterion not met

Publication bias: (5a) asymmetrical funnel plot; (5b) flaws in literature search

Confidence ratings reported from qualitative studies, assessed using the GRADE-CERQual tool, are shown in Table 5. Of the 18 outcomes, three provided moderate-quality confidence (16.7%), six provided low-quality confidence (33.3%), and nine provided very low-quality confidence (50.0%). The general reasons for downgrading of ratings were as follow: (1) We downgraded the confidence ratings based on common methodological limitations included incomplete reporting of random sequence generation and allocation concealment, no blinding, failure to adhere to the intention-to-treat, and including non-RCTs. (2) The data were often assessed as being either poor or unclear coherence, mainly because of conflicting results or limited data, respectively. (3) Our concerns about adequacy were mainly tied to the small number of articles and small sample sizes within available studies.

**Table 5:** Summary of qualitative findings

Outcomes	Study	Overall CERQual assessment	Explanation for assessment	Contributing studies
Reduction in limb circumference (LLLT versus placebo laser at 1-month follow-up)	Baxter (2017)	Low confidence	Methodological limitations: Three studies have moderate methodological limitations; Relevance: High Coherence: High Adequacy of data: The research covers 1 developed country and 2 developing countries. The information is relatively single and limited	Omar (2011) Kaviani (2006) Carati (2003)
Reduction in limb circumference (LLLT versus conventional therapy at short-term follow-up]	Baxter (2017)	Very low confidence	Methodological limitations: Three studies have moderate to severe methodological limitations Relevance: High Coherence: Conflicting results leads to poor consistency Adequacy of data: The research covers 1 developed country and 2 developing countries. The information is relatively single and limited	Rinder (2013) Kozanoglu (2009) Maiya (2008)
Reduction in limb volume	E Lima (2014)	Moderate confidence	Methodological limitations: Four studies have moderate to severe methodological limitations Relevance: High Coherence: High Adequacy of data: The research covers 1 developed country and 3 developing countries. The information is relatively sufficient	Lau (2009) Kozanoglu (2009) Kaviani (2006) Carati (2003)
Reduction in limb volume	Moseley (2007)	Low confidence	Methodological limitations: Three studies have moderate to severe methodological limitations Relevance: High Coherence: High Adequacy of data: The research covers 2 developed countries. The information is relatively single and limited	Carati (2003) Piller (1998) Piller (1995)
Reduction in limb volume (LLLT versus placebo laser at post-treatment)	Baxter (2017)	Very low confidence	Methodological limitations: Two studies have moderate methodological limitations Relevance: High Coherence: Conflicting results leads to poor consistency Adequacy of data: The research covers 1 developed country and 1 developing country. The information is relatively single and limited	Omar (2011) Carati (2003)
Reduction in limb volume (LLLT versus no treatment at 1-month follow-up)	Baxter (2017)	Very low confidence	Methodological limitations: One study has high methodological limitation Relevance: High Coherence: Limited data results in unclear consistency Adequacy of data: The research covers 1 developing country. The information is relatively single and limited	Lau (2009)
Reduction in limb circumference or volume	Monteiro (2014)	Moderate confidence	Methodological limitations: Five studies have moderate to severe methodological limitations Relevance: High Coherence: High Adequacy of data: The research covers 2 developed countries and 3 developing country. The information is relatively sufficient	Rinder (2013) Omar (2011) Lau (2009) Kaviani (2006) Carati (2003)
Reduction in limb circumference or volume	Omar (2012)	Moderate confidence	Methodological limitations: Five studies have moderate to severe methodological limitations Relevance: High Coherence: High Adequacy of data: The research covers 1 developed country and 4 developing countries. The information is relatively sufficient	Omar (2011) Lau (2009) Kozanoglu (2009) Kaviani (2006) Carati (2003)
Improvement of subjective symptoms	E Lima (2014)	Low confidence	Methodological limitations: Four studies have moderate to severe methodological limitations Relevance: High Coherence: Conflicting results leads to poor consistency Adequacy of data: The research covers 1 developed country and 3 developing countries. The information is relatively sufficient	Lau (2009) Kozanoglu (2009) Kaviani (2006) Carati (2003)
Improvement of subjective symptoms	Moseley (2007)	Low confidence	Methodological limitations: Three studies have moderate to severe methodological limitations Relevance: High Coherence: High Adequacy of data: The research covers 2 developed countries. The information is relatively single and limited	Carati (2003) Piller (1998) Piller (1995)
Pain relief	Monteiro (2014)	Very low confidence	Methodological limitations: Three studies have moderate to severe methodological limitations Relevance: High Coherence: Conflicting results leads to poor consistency Adequacy of data: The research covers 1 developed country and 2 developing countries. The information is relatively single and limited	Lau (2009) Kaviani (2006) Carati (2003)
Pain relief (LLLT versus placebo laser at 2-month follow-up)	Baxter (2017)	Very low confidence	Methodological limitations: One study has moderate methodological limitation Relevance: High Coherence: Limited data results in unclear consistency Adequacy of data: The research covers 1 developing country. The information is relatively single and limited	Kaviani (2006)
Pain relief (LLLT versus conventional therapy at post-treatment)	Baxter (2017)	Very low confidence	Methodological limitations: Two studies have moderate methodological limitations; Relevance: High Coherence: Conflicting results leads to poor consistency Adequacy of data: The research covers 2 developing countries. The information is relatively single and limited	Kozanoglu (2009) Maiya (2008)
Pain relief (LLLT versus conventional therapy at 3-month post-treatment)	Baxter (2017)	Very low confidence	Methodological limitations: One study has moderate methodological limitation; Relevance: High Coherence: Limited data results in unclear consistency Adequacy of data: The research covers 1 developing country. The information is relatively single and limited	Kozanoglu (2009)
Improvement of quality of life;	Moseley (2007)	Very low confidence	Methodological limitations: One study has moderate methodological limitation; Relevance: High Coherence: Limited data results in unclear consistency Adequacy of data: The research covers 1 developed country. The information is relatively single and limited	Carati (2003)
Shoulder mobility (LLLT versus placebo laser at post-treatment)	Baxter (2017)	Very low confidence	Methodological limitations: Two studies have moderate methodological limitations; Relevance: High Coherence: Conflicting results leads to poor consistency Adequacy of data: The research covers 1 developed country and 1 developing country. The information is relatively single and limited	Omar (2011) Carati (2003)
Range of movement in the affected limb [LLLT versus placebo laser at short-term follow-up (< 6 months)]	Baxter (2017)	Low confidence	Methodological limitations: Two studies have moderate methodological limitations; Relevance: High; Coherence: High Adequacy of data: The research covers 1 developed country and 1 developing country. The information is relatively single and limited	Kaviani (2006) Carati (2003)
Decrease in tissue hardness	E Lima (2014)	Low confidence	Methodological limitations: Two studies have moderate methodological limitations; Relevance: High; Coherence: High Adequacy of data: The research covers 1 developed country and 1 developing country. The information is relatively single and limited	Lau (2009) Carati (2003)

*CERQual*, Confidence in the Evidence from Reviews of Qualitative research; *LLLT*, low-level laser therapy

**Table 6:** Main characteristics of included randomized controlled trials

Author (year)	Inclusion criteria	No. of patients	Intervention group	Control group	Co-intervention	Assessment times	Outcomes reported
Kilmartin (2020)	1) Woman aged ≥ 21 year; 2) Diagnosis of BCRL (girth ≥ 2 cm circumferential difference and/or volume ≥ 200 mL compared with the uninvolved upper extremity at any 4 cm segment) 3) Stage II or III lymphedema (as defined by the International Society of Lymphology)	L:11 C:10	**CDT + active LLLT** (2 times/w × 8–16 sessions with 1.5 J/cm^2^, 1 min/10 sites in the axilla and a portion of the chest wall on the affected side)	**CDT + inactive laser**	NR	Pre-treatment; post-treatment (8 sessions); post-treatment (16 sessions); 3-week follow-up; 6-week follow-up; 12-week follow-up	Lymphedema symptoms, symptom distress, limb volume
Baxter (2018)	1) Woman aged over 18 year; 2) Diagnosis of BCRL (defined as a circumference increase over 7.5% at any measurement level in the operated arm compared with the control)	L:9 C:8	**Conventional therapy + LLLT** (2 times/w × 6 w with 6 J/cm^2^, wavelength with 980/810 nm; output power with 500 mW beam spot size 5 cm^2^; irradiance with 100 mW/cm^2^, 1 min/10 sites from axilla to wrist)	**Conventional therapy** (continuous wearing of a pressure garment, massage therapy, and remedial exercises, e.g., aerobic exercise, strength training, and stretching exercises)	NR	Pre-treatment; post-treatment 6-week follow-up	Limb circumference differences; participant’s perceived symptoms (pain and heaviness); psychological impacts (self-consciousness, anxiety, perception of arm swelling, and emotion changes); activity disability
Storz (2017)	≧ 3 months history of PML (either modified radical mastectomy or breast-conserving surgery with axillary dissection or sentinel lymph node biopsy)	L:20 C:20	**Active laser** (2 times/w × 4 w with 4.89 J/cm^2^, wavelength with 980 nm; total power output with 640 mW; 10 min/session, over the whole axillary)	**Placebo laser**	Daily limb exercises; skin care	Pre-treatment post-treatment 4-week follow-up; 8-week follow-up; 12-week follow-up	Lymphedema-related pain; quality of life; grip strength; limb volume difference
Ridner (2013)	1) Age≧ 21 year 2) Stage I or II lymphedema as determined by a physician and defined by the International Society of Lymphology (1995)	L: 15 M:16 L + M:15	**LLLT alone (**20 ~ 30 s/point in each grid, 20 min/session) **MLD + LLLT (**each session 20 min of LLLT + 20 min of MLD);	**MLD alone** (40 min/session)	Compression bandaging after treatment	Pre-treatment; post-treatment	Limb volume (circumferential measurement); extracellular fluid (bioelectrical impedance); psychological and physical symptoms; quality of life
Omar (2011)	1) Stage II or III lymphedema 2) Diagnosis of BCRL (an increase in arm circumference at any level by 2 cm and less than 8 cm compared with the contralateral side)	L:25 C:25	**Active LLLT** (3 times/w × 12 w with 1.5 J/cm^2^, wavelength with 904 nm; power with 5 mW; seven points over the axillary and arm areas)	**Placebo sham**	Limb exercise Skin care instructions Wear pressure garment	Pre-treatment; post-treatment; 4-week follow-up	Limb circumference difference; shoulder mobility; grip strength
Lau (2009)	1) Women aged ≧ 18 year had undergone unilateral standard or modified radical mastectomy with subsequent radiotherapy or chemotherapy 2) Diagnosis of BCRL (more than 200 mL difference between arms)	L:11 C:10	**LLLT:** 3 times/w × 4 w with 2 J/cm^2^, wavelength with 905 nm, output with 24 mW, frequency varying from 1 to 10,000 Hz; wavelength with 808 nm, output with 500 mW, frequency varying from 1 to 1500 Hz; 20 min/session)	**No treatment**	Lymphedema education session	Pre-treatment; post-treatment; 4-week follow-up	Limb volume; tissue resistance subjective symptoms [Disabilities of Arm, Shoulder, and Hand (DASH) questionnaire]
Kozanoglu (2009)	1) ≧3 months history of arm lymphedema 2) Diagnosis of lymphedema (a difference of more than 2 cm at at least three of the seven points, including the axilla, 10 cm proximal and distal to the antecubital fossa, elbow, 5 cm proximal to the wrist, wrist and midpalm)	L:25 C:25	**LLLT** (3 times/w × 4 w with 1.5 J/cm^2^; 20 min/session; wavelength with 904 nm; treatment area over three points on the antecubital fossa and at seven points on the axilla)	**Pneumatic compression therapy** (2 h with pressure of 60 mmHg)	Daily limb exercises (active range of motion, elevation and pumping exercises); hygiene; skin care	Pre-treatment; post-treatment; 3-month follow-up; 6-month follow-up; 12-month follow-up	Difference between sum of the circumferences of affected and unaffected limbs; pain; range of motion of the upper extremity joints; grip strength
Maiya (2008)	Women with unilateral BCRL (> 2-cm interlimb difference at 2 sites)	L:10 C:10	**LLLT** (34 min/session, daily for 10 days, wavelength with 632.8 nm and 850 nm; 2.4 J/cm^2^)	**Compression bandage**	Upper extremity exercise program	Pre-treatment; post-treatment	Limb circumference Pain
Kaviani (2006)	1) **≧** 3 months history of unilateral arm lymphedema in women after modified radical mastectomy and received radiation therapy 2) Arm lymphedema was defined as 2 cm or more difference in circumference between the two arms at midhumeral line	L:6 C:5	**LLLT:** 2 blocks (3 times/w × 3 w with 1.5 J/cm^2^, wavelength with 890 nm; output Power with 10 W; over the five points applied to the axillary region) separated by 8 w rest period	**Placebo laser**	NR	Pre-treatment; during the treatment (weeks 3, 9, 12, 18, and 22)	limb circumference difference; pain; range of motion; heaviness; desire to continue the treatment
Carati (2003)	1) women aged ≧ 18 years 2) diagnosis of clinically manifest PML (> 200 mL difference between arms or ≥ 2 cm difference in arm circumference at ≥ 3 positions)	L:33 C:28	**2 blocks of active LLLT** (3 times/w × 3 w with 1.5 J/cm^2^, wavelength with 904 nm; power with 5 mW from a treatment head measuring 0.2 cm^2^ in size; 1 min/17 treatment points) separated by 8 w rest period	**1 block of sham therapy** followed by 8 w rest period then **1 block of active LLLT** (3 times/w × 3 w with 1.5 J/cm/^2^)	NR	Pre-treatment; post-treatment: 1-month follow-up; 2-month follow-up; 3-month follow-up	Limb volume; bioimpedance; tissue resistance; shoulder range of movement; self-report (perceptual symptoms of their affected limb, the ability to perform specific activities of daily living, overall feelings regarding quality of life)

*BCRL*, breast cancer–related lymphedema; *CDT*, complex decongestive therapy; *LLLT*, low-level laser therapy; *PML*, postmastectomy lymphedema; *MLD*, manual lymphatic drainage; *NR*, not reported; *min*, minutes; *w*, week

#### Effectiveness of LLLT

The conclusions were divergent on treatment effect (limb circumference reduction, limb volume reduction, tissue hardness, subjective symptoms, grip strength, and quality of life). There is no consensus regarding the effectiveness of LLLT is significant and clear. For instance, Chen et al. [25] found no differences between groups for limb circumference reduction. In contrast, the reviews from Smoot et al. [27], Lima et al. [29], and Omar et al. [30] found LLLT was superior to control group in limb volume reduction. Baxter et al. [26] provided conflicting evidence regarding the effects of LLLT over sham laser on limb volume reduction.

### Updated systematic review

#### Search results

**Fig. 3:**
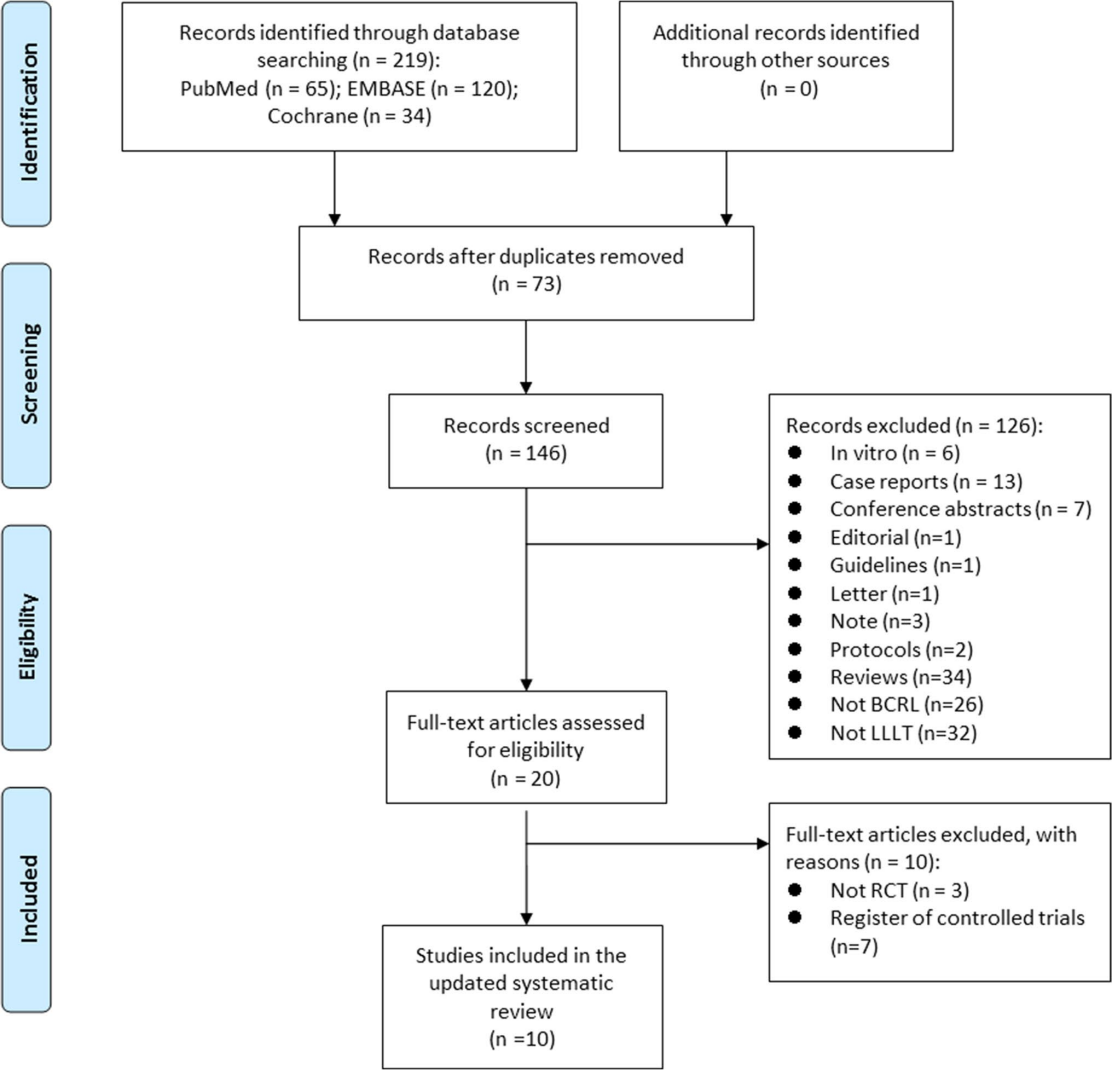
PRISMA flow diagram illustrating the selection of randomized controlled trials. *BCRL* breast cancer–related lymphedema, *LLLT* low-level laser therapy, *RCTs* randomized controlled trials

We identified a total of 219 records through three electronic databases. Of the 146 records remaining after duplicate removal, we excluded 125 based on the information in the title and/or abstract. We retrieved the full papers for the remaining 20 citations. After full-text review, we excluded 10 papers and these are listed with the reasons in the Supplemental Table 3. The PRISMA flow diagram of selected RCTs is illustrated in Fig. 3.

#### Study characteristics

The main characteristics of the included RCTs are listed in Table 6. The publication dates of the studies were between 2003 and 2020, and the sample sizes of these studies ranged from 11 to 61 female patients. The eight included trials had definite diagnostic criteria for BCRL with volume difference greater than 200 ml between limbs [32, 33, 45], or circumference difference greater than 2 cm between limbs [32, 34–36, 39, 45], or circumference increase over 7.5% between limbs [42]. The two trials compared LLLT alone with some form of compression therapies (pneumatic compression [36], and compression bandage [39]). One trial [37] conducted the comparison among LLLT alone, MLD alone, and combined LLLT and MLD. One trial combined LLLT with conventional therapy [42] as the intervention group. One [45] compared active LLLT plus CDT with inactive laser plus CDT. Only one trial [33] designed their control group as not receiving any treatment, and the other four trials [32, 34, 35, 43] compared the outcomes of LLLT with placebo laser. All trials assessed limb circumference/volume reduction as the primary outcome. Outcome measures were collected at pre-treatment and immediately post-treatment in all trials. Follow-up periods differed between studies, with most trials reporting a follow-up period of 3 months or less. One trial [36] reported outcome measures over a longer follow-up period, namely 12 months.

#### Assessment of risk of bias for primary trials and quality of evidence

An overview of the risk of bias for included primary trials is presented in Supplemental Fig. 1 and 2. We deemed eight studies [32, 33, 35, 37, 39, 42, 43, 45] to be at low risk of bias, and two studies [34, 36] at unclear risk of bias for random sequence generation. We judged random sequence allocation and allocation concealment as sufficient in only two trials [36, 42], whereas the other trials [32–35, 37, 39, 43, 45] did not specify allocation methodology to be at unclear risk of bias. Blinding participants would have only been possible had there been a placebo laser therapy compared with a real LLLT: six studies [32–35, 43, 45] showed a low risk and four [36, 37, 39, 42] for high risk of bias. Five trials [30, 34, 42, 43, 45] did not blind the outcomes assessor and were therefore judged as having a high-risk of bias; and the other five trials [32, 33, 36, 37, 39] clearly blind the outcomes assessor and were regarded as low risk of bias. Incomplete outcome data examined the number of drop-outs and found two studies [34, 45] had a high risk, the other trials [32, 33, 35–37, 39, 42, 43] at low risk of bias. All studies [32–37, 39, 42, 43, 45] did not publish a protocol paper and were therefore considered to be at unclear risk of bias regarding selective reporting. We also considered fund support and conflicts of interest as potential sources of bias. GRADE results deemed all outcomes in included primary trials to be at low-quality evidence due to risk of bias and imprecision.

#### Effectiveness of LLLT

**Fig. 4:**
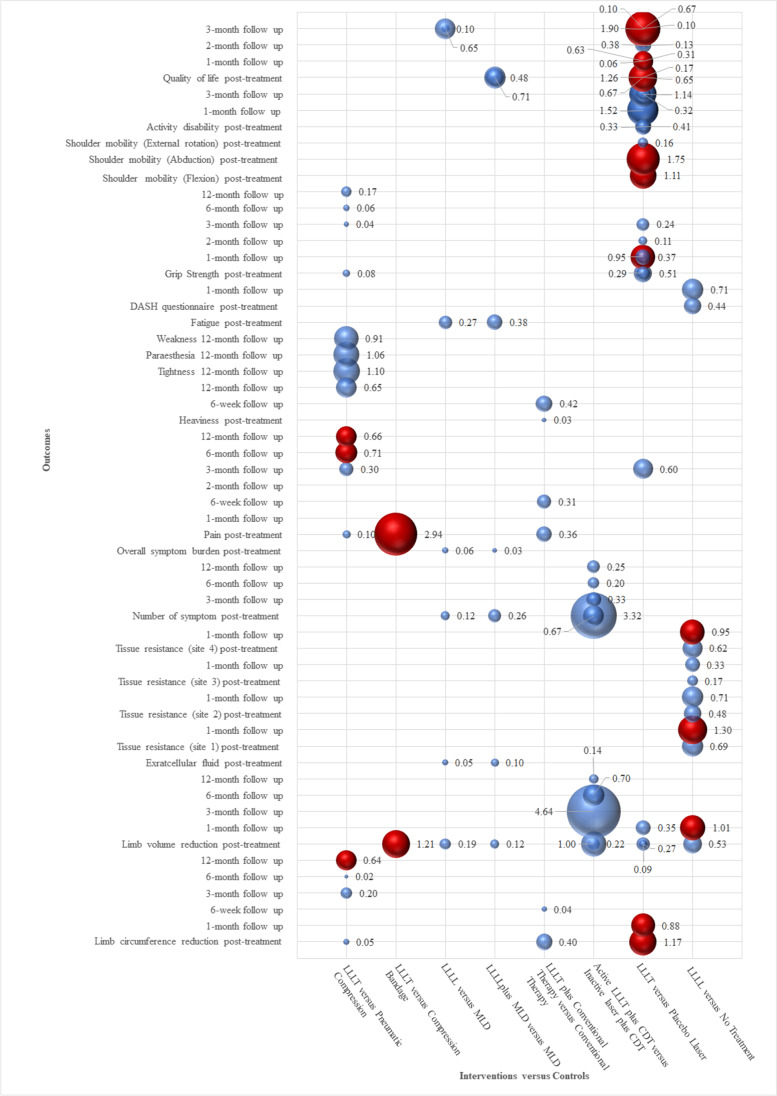
The bubble plot regarding to all outcomes at different comparison categories for management of breast cancer related lymphedema. The x-axis represented seven comparison categories in all trials. The y-axis represented each clinical outcome at different assessment times. The bubble size represented effectiveness estimate of each outcome. The different colors represented statistical differences (red bubbles indicated that the difference was statistically significant, blue bubbles indicated that the difference was not statistically significant). *LLLT* low-level laser therapy, *MLD* manual lymphatic drainage, *CDT* complex decongestive therapy, *DASH* Disability of Arm, Shoulder, and Hand

The effects of the intervention (LLLT) for each outcome analyzed are presented below. Results are discussed separately depending on the type of comparison (control) (Supplemental Table 4). The bubble plot was created to present evidence for each outcome with different comparison categories (Fig. 4).

##### Comparison 1: LLLT versus pneumatic compression

Only one trial [36] reported on this comparison.

**Limb circumference reduction**. LLLT significantly reduced limb circumference compared to pneumatic compression at 12-month follow-up (*SMD* = 0.64, 95% CI: 0.05 to 1.22). There were no difference between-groups at immediately post-treatment, 3-month follow-up and 6-month follow-up.

**Pain**. LLLT showed a statistically significant benefit favoring pneumatic compression in pain reduction at 6-month follow-up (*SMD* = 0.71, 95% CI: 0.12 to 1.30), and 12-month follow-up (*SMD* = 0.66, 95% CI: 0.07 to 1.25). There were no difference between-groups differences at immediately post-treatment and 3-month follow-up.

**Grip strength**. There was no significant difference in the improvement of grip strength between groups (either immediately post-treatment or follow-up periods).

**Subjective symptoms**. There was no significant difference in the improvement of subjective symptoms (heaviness, tightness, paraesthesia, and weakness) between the groups at 12-month follow-up.

##### Comparison 2: LLLT versus compression bandage

Only one trial [39] reported on this comparison.

**Limb circumference reduction.** The result showed a significant benefit of LLLT at immediately post-treatment as compared to compression bandage (*SMD* = 1.21, 95% CI: 0.25 to 2.15).

**Pain.** The result suggested that LLLT was more effective for pain relief at immediately post-treatment, when compared to compression bandage (*SMD* = 2.94, 95% CI: 1.68 to 4.21).

##### Comparison 3: LLLT versus MLD versus combined LLLT and MLD

Only one trial [42] reported on this comparison. No statistically significant differences in limb volume reduction, extracellular fluid reduction, subjective symptoms (symptom number, burden, or fatigue) and quality of life [the Profile of Mood States–Short Form (POMS-SF) and Upper Limb Lymphedema-27 (ULL-27)] among the groups were observed at immediately post-treatment.

##### Comparison 4: LLLT plus conventional therapy versus conventional therapy

Only one trial [42] reported on this comparison. No statistically significant differences in limb circumference reduction, subjective symptoms (pain and heaviness), and activity disability (put on bra, tie shoe laces, wash hair, and hang out washing) between the groups were observed at immediately post-treatment and 6-week follow-up. It is important to highlight that due to no improvement of activity disability in control group, we cannot calculate SMD and 95% CI between groups.

##### Comparison 5: active LLLT plus CDT versus inactive laser plus CDT

Only one trial [45] reported on this comparison. No statistically significant differences in limb volume reduction, and the number lymphedema symptoms when active LLLT plus CDT with inactive laser plus CDT (either immediately post-treatment or follow-up periods).

##### Comparison 6: LLLT versus placebo laser

Four trials [32, 34, 35, 43] fit this comparison category. Since data cannot be extracted from one trial [34], we cannot calculate the corresponding effect size and 95% CI and only analyzed the other three trials.

**Limb circumference reduction.** One pooled trial [35] showed LLLT significance favoring placebo laser at immediately post-treatment (*SMD* = 1.17, 95% CI: 0.59 to 1.75) and 12-month follow-up (*SMD* = 0.88, 95% CI: 0.32 to 1.44).

**Limb volume reduction***.* The two trials [32, 43] that reported on this outcome found no statistically significant differences between the groups (either immediately post-treatment or 1-month follow-up).

**Pain***.* One pooled trial [43] reported that the effect of LLLT on pain relief did not significantly differ from placebo laser at the end of the treatment and subsequent follow-up periods.

**Grip strength***.* Of the two trials that reported on this outcome, there were no significant between-groups differences at either immediately post-treatment or follow-up periods in one trial [43]. The other trial [35] reported similar result at post-treatment, yet positive result at 1-month follow-up that more significant improvement of grip strength was observed in LLLT group than placebo laser group (*SMD* = 0.95, 95% CI: 0.39 to 1.95).

**Shoulder mobility***.* At immediately post-treatment, there was statistically significant improvement in shoulder mobility (flexion and abduction) for the LLLT compared with placebo group (*SMD* = 1.11, 95% CI: 0.53 to 1.68; *SMD* = 1.75, 95% CI: 1.12 to 2.38), while shoulder external rotation showed no statistically significant differences at any point of evaluation between two groups in one trial [35].

**Activity disability***,* One trial [32] reported that there was no difference in activities of daily living (ability to put on bra, tie shoes, wash hair, hang out washing) found between two groups.

**Quality of life***.* Of the two trials that reported on this outcome, there were no significant between-groups differences at either immediately post-treatment or follow-up periods in one trial [43]. While, the other trial [32] demonstrated that there was a significant improvement in the quality of life after 1 cycle of LLLT at immediately post-treatment (*SMD* = 0.67, 95% CI: 0.15 to 1.19) and 3-month follow-up (*SMD* = 0.67, 95% CI: 0.15 to 1.19). Similar results were observed after 2 cycles of LLLT compared with placebo group at immediately post-treatment (*SMD* = 1.26, 95% CI: 0.67 to 1.86), 1-month follow-up (*SMD* = 0.63, 95% CI: 0.07 to 1.19), and 3-month follow-up (*SMD* = 1.90, 95% CI: 1.24 to 2.56).

##### Comparison 7: LLLT versus no treatment

Only one trial [33] reported on this comparison.

**Limb volume reduction***.* At 1-month follow-up, the laser group had a 28% cumulative reduction in the limb volume in contrast to a 6% increase in the control group. The between-group difference reached significance level (*SMD* = 1.01, 95% CI: 0.09 to 1.92).

**Tissue resistance***.* At 1-month follow-up, there was a 33.2% cumulative increase in tonometry reading at site 1 and a 15.2% cumulative increase at site 4 in LLLT group, while only negligible changes in the control group. Significant between-group difference was found at sites 1 and 4 (*SMD* = 1.30, 95% CI: 0.35 to 2.25; *SMD* = 0.95, 95% CI: 0.04 to 1.85).

**Subjective symptoms**. At the end of the treatment and at subsequent follow-up assessments, the LLLT group demonstrated a 20%/37% cumulative reduction in the Chinese version of the Disability of Arm, Shoulder, and Hand (DASH) scores, compared to a 1%/7% cumulative increase in DASH scores for the control group. Although the LLLT group tended to show a greater reduction, the between-group differences in the mean DASH scores were not statistically significant.

#### Safety of LLLT

Four trials focused on adverse events. Baxter et al. [27] reported one participant experienced cellulitis in her affected arm after the 6th treatment session. The other three trials [32, 34, 43] suggested there were no adverse reactions or side effects reported among any participants.

## Discussion

Nowadays, it is commonly considered that systematic reviews and meta-analyses can provide more reliable evidence than individual trials, as their outcomes are derived from all published RCTs and as they can be systematically reviewed for the risk of bias [46]. The rationale for choosing to perform an overview of systematic reviews is in supporting a faster and more reliable decision-making for the clinician.

### Overview of systematic reviews

#### Summary of main results

This overview of seven systematic reviews summarized the clinical evidence on the effectiveness and safety of LLLT for BCRL. Although most studies have demonstrated efficacy in management of BCRL, not all reviews have yielded positive outcomes. Therefore, the result that LLLT appears to be superior to other therapies has not been clearly established in our overview.

#### Interpretation of findings

As discussed below, these divergent results may be attributed to several factors. An important aspect that has to be mentioned is the type of control groups. Because it is possible that LLLT may offer the same results as conventional therapies and that the combination of therapies offers no additional benefit, it may show greater effect than placebo laser therapy or no treatment or wait list. Clinical appropriateness of pooling study results irrespective of control comparisons (lack of subgroup analysis) may limit the validity of the review conclusions.

Perhaps, extensive heterogeneity in laser therapy regimens (wavelength, dosage, duration, frequency, and emitting zone) was responsible for inconsistent conclusions. Different laser therapy parameters may have different biological regulation effects. The biological regulation of laser therapy depends on the absorption of light by the chromophore. Each chromophore will only absorb the photon with in a specific wavelength range. However, even with a compatible wavelength, the cellular effect varies in intensity according to the amount of energy supplied [47]. There is a biphasic dose response curve in which low dose is not enough to trigger significant biological effect, while excessive light delivery can lead to unwanted inhibitory effects [48, 49]. From our overview, infrared wavelengths between 808 and 905 nm have been most commonly employed to date, and energy densities in the range of 1.5–2.4 J/cm^2^ have delivered positive outcomes. The application of high energy densities (4.89 and 6 J/cm^2^) might exert opposite effects due to tissue destruction rather than healing [43]. The laser application duration varied from 17 to 34 min per session; the frequency varied from ten sessions on consecutive days to 36 sessions provided 3 times/week for 12 weeks. The emitting zone is also crucial to the effect of therapy. Because of the nonuniformity of the irradiated anatomical area, all studies had their own emitting areas, ranging from 5 to 17 spots including axillary region, and arm region, which may have directly affected the total energy received [35, 36]. To achieve positive results, a well-conceived therapy schedule and an adequate laser configuration are necessary.

The measurement methods and assessment times of outcomes may be a critical influence factor for heterogeneity of research results. For instance, limb volume is measured by different methods across studies: water displacement (volumetry) [33], truncated cone method [32], circumference measurements [37, 43]. As the change of assessment time, the effectiveness may be different due to disease progression or recurrence. It is worth considering whether these outcome measures are similar in structure and whether they are appropriate to be combined in pooled analysis.

#### Assessment of quality

From this overview, we found that the methodological quality and evidence quality of included reviews were unsatisfactory. According to AMSTAR-2, the methodological quality of all included reviews was low or critically low. The most common of domains not addressed were no prior registration, no excluded studies list, and no reporting on funding sources. The lack of registration may result in a great adjustment of the study process than expected, increasing the risk of bias and affecting the rigor of the reviews. The lack of excluded studies lists with reasons, which may undermine the transparency of the reviews and affect the reliability of their results. The lack of report on funding sources may reduce the credibility of the research results due to potential conflicts of interest. Overall, the studies included in this review were of a suboptimal quality, which in turn affects our assessment of the certainty of the evidence available from the analyzed results.

According to GRADE and GRADE-CERQual, evidence quality of outcome measurement was between moderate and very low. Most outcomes were with low or very low-quality evidence. The main limitations are imprecision associated with suboptimal sample sizes and risk of bias associated with poor reporting of study methods. Most studies failed to describe methods of randomization or to provide sufficient details about blinding or allocation concealment. Given the poor quality of the quantitative evidence, it is uncertain regarding effectiveness of LLLT. The existing systemic reviews with meta-analyses do not provide clear guidance for clinical practice in this area. It highlights the need for high-quality RCTs to establish firm conclusions.

### Updated systematic review

Due to the fact that the overview has not reached a unified conclusion, we further reviewed the results of ten clinical trials assessing the relative contribution of LLLT in treating BCRL. Several outcomes have been examined including the objective outcomes of limb circumference, limb volume reduction, extracellular fluid, tissue resistance, grip strength, and shoulder mobility as well as the subjective outcomes of pain, heaviness, tightness, paraesthesia and weakness, activity disability, and quality of life.

Our updated systematic review found that LLLT results in greater improvements compared to compression therapy, placebo laser, or no treatment, and appears to be relatively safe. Nevertheless, many trials had a high or unclear risk of bias for two or more items. In addition, GRADE result showed low-quality of evidence per outcome. Therefore, these results should be interpreted with caution. We are uncertain to reach these conclusions that LLLT is superior to another active or negative intervention at short-term and long-term and LLLT is safe. More RCTs of high methodological quality, with large sample sizes and long-term follow-up, are needed to inform clinical guidelines and routine practice.

In addition, we found there were no statistically significant differences regarding our outcome variables between LLLT and CDT, MLD, or conventional therapy over the time course. There are two possible explanations for the similar effects: chance or a true finding. If the results are due to chance, then future attempts to replicate these results would most likely show a different finding. If the results are due to a true finding, LLLT may have the same effects as aforementioned therapies and that the combination of therapies offers no additional benefit. LLLT may offer a time saving therapeutic option to CDT, MLD, or conventional therapy.

### Strengths and limitation

This overview has several strengths. Firstly, to be the best of our knowledge, this is the first overview to explore the effectiveness and safety of LLLT in treatment of BCRL. Secondly, we assessed the methodological quality of included reviews using the AMSTAR 2 tool, and we assessed clinical outcomes using the GRADE or GRADE-CERQual score to determine strength of evidence. Thirdly, we updated the systematic review; subgroup analyses stratified by control comparisons were performed to address the influence of clinical (as well as statistical) heterogeneity.

Nonetheless, there are some limitations worth mentioning in this overview: (1) a potential limitation was the heterogeneity across trials (e.g., different comparison mode). To solve this potential limitation, we conducted comparison modes subgroup analyses to explore their difference. In our updated systemic review, we classified 10 RCTs into seven comparisons, only one study in most comparisons. Even compared with the placebo laser in four trials, each outcome included only one or two trials. Although some positive results have been found, we cannot draw definitive conclusions regarding the effectiveness of LLLT in patients with BCRL due to small number of included trials. (2) The poor quality of the included primary trials, and evidence quality of outcomes was unsatisfactory, which hinder the possibility of any solid conclusion. (3) Age and time of diagnosis could interfere with results of studies. The older the age and the later the diagnosis, the more likely the function of the lymphatic system may be poor. However, to our knowledge, there has been no any study investigating the effects of age or time of diagnosis on results. (4) It is variability in the numerical reporting of the results of the RCTs included in our updated review. In some cases, these data were only reported graphically without the corresponding raw data. Even using specialized software (e.g., GetData Graph Digitizer 2.26), we still cannot retrieve the original data from graphs. In some cases, some reported data were incomplete, particularly continuous variables where the mean of the change (final versus basal) and its SD were required. We need to transform the data. This issue is particularly relevant in this review since the original studies were mostly small trials, and despite randomization there were baseline differences between the groups in most of the variables analyzed. This shortcoming has forced us to combine results in our analysis where effects have been quantified using different methods, which adds uncertainty to the results obtained.

## Conclusion

Due to insufficient data and poor quality of evidence, there is uncertain to reach these conclusions that LLLT is superior to another active or negative intervention and is safe. More RCTs of high methodological quality, with large sample sizes and long-term follow-up, are needed to inform clinical guidelines and routine practice.

## Supplementary Information

Below is the link to the electronic supplementary material.

Supplementary file1 (DOCX 58 KB)

## Data Availability

All data generated for this review are included in the manuscript and/or the supplementary files.
