# Editorial Expression of Concern: The effectiveness and safety of low‑level laser therapy on breast cancer–related lymphedema: An overview and update of systematic reviews

**DOI:** 10.1007/s10103-023-03815-0

**Published:** 2023-07-04

**Authors:** Yuping Wang, Yonggui Ge, Wenting Xing, Junping Liu, Jiqi Wu, Haijuan Lin, Yaqin Lu

**Affiliations:** Department of Rehabilitation, The First Hospital of Lanzhou University, No. 1 Donggang West Road, Chengguan District, Lanzhou, 730000 People’s Republic of China

**Editorial Expression of Concern: Lasers Med Sci**

10.1007/s10103-021-03446-3

After publication of this article [1], a reader alerted the Editor-in-Chief that the results of a trial by Carati et al. (reference 32) [2] have been presented inaccurately in the review article. The authors reported no statistically significant results after 1 round of treatment, but didn't mention the significant results after 2 rounds of treatment and with longer follow up. The authors did not respond to the request for an explanation or correction. Readers are advised to interpret the results with caution.

The authors have not responded to any correspondence from the editor or publisher about this Editorial Expression of Concern.
