# Supplementary material for: The effectiveness and safety of low-level laser therapy on breast cancer–related lymphedema: An overview and update of systematic reviews
Source: Lasers Med Sci. 2021 Nov 15;37(3):1389–413. doi: 10.1007/s10103-021-03446-3 (PMC8971164; doi:10.1007/s10103-021-03446-3)

**Supplemental Table 1 Search strategy via Pubmed as an example**

| Search number | Query |
| --- | --- |
| #1 | "Breast Neoplasms"[Mesh] |
| #2 | (breast cancer*[Title/Abstract]) OR (breast neoplasm*[Title/Abstract]) OR (breast carcinoma*[Title/Abstract]) OR (breast tumour*[Title/Abstract]) OR (breast tumor*[Title/Abstract]) OR (Mammary Cancer*[Title/Abstract]) OR (Mammary Carcinoma*[Title/Abstract]) OR (Mammary Neoplasm*[Title/Abstract]) OR (Breast Malignant Neoplasm*[Title/Abstract]) OR (Breast Malignant Tumor*[Title/Abstract]) |
| #3 | #1 OR #2 |
| #4 | "Lymphedema"[Mesh] |
| #5 | (lymphedem*[Title/Abstract]) OR (lymphoedem*[Title/Abstract]) OR (oedema* [Title/Abstract]) OR (edema*[Title/Abstract]) OR (swelling[Title/Abstract]) OR (elephantiasis[Title/Abstract]) OR (dropsy[Title/Abstract]) OR (hydrops[Title/Abstract]) |
| #6 | #4 OR #5 |
| #7 | #3 AND #6 |
| #8 | "Breast Cancer Lymphedema"[Mesh] |
| #9 | (Breast Cancer Lymphedema*[Title/Abstract]) OR (Breast Cancer Treatment-Related Lymphedema[Title/Abstract]) OR (Breast Cancer Treatment Related Lymphedema[Title/Abstract]) OR (Breast Cancer-Related Arm Lymphedema[Title/Abstract]) OR (Breast Cancer Related Arm Lymphedema[Title/Abstract]) OR (Breast Cancer Related Lymphedema[Title/Abstract]) OR (Postmastectomy Lymphedema*[Title/Abstract]) OR (Post-mastectomy Lymphedema*[Title/Abstract]) OR (Post mastectomy Lymphedema*[Title/Abstract]) |
| #10 | #8 OR #9 |
| #11 | #7 OR #10 |
| #12 | "Low-Level Light Therapy"[Mesh] |
| #13 | (Low Level Light Therapy[Title/Abstract]) OR (Low-Level Light Therapies[Title/Abstract]) OR (Photobiomodulation Therapy[Title/Abstract]) OR (Photobiomodulation Therapies[Title/Abstract]) OR (Low Level Laser Therapy[Title/Abstract]) OR (Low-Level Laser Therapy[Title/Abstract]) OR (Low-Level Laser Therapies[Title/Abstract]) OR (Low Power Laser Therapy[Title/Abstract]) OR (Low-Power Laser Therapy[Title/Abstract]) OR (Low-Power Laser Therapies[Title/Abstract]) OR (Low-Power Laser Irradiation[Title/Abstract]) OR (Low Power Laser Irradiation[Title/Abstract]) OR (Laser Biostimulation[Title/Abstract]) OR (Laser Phototherapy[Title/Abstract]) OR (LLLT[Title/Abstract]) |
| #14 | "Laser Therapy"[Mesh] |
| #15 | (Laser Therapy[Title/Abstract]) OR (Laser Therapies[Title/Abstract]) |
| #16 | "Lasers"[Mesh] |
| #17 | (Laser[Title/Abstract]) OR (Lasers[Title/Abstract]) |
| #18 | #12 OR #13 OR #14 OR #15 OR #16 OR #17 |
| #19 | "Meta-Analysis as Topic"[Mesh] OR (Meta-Analysis [Publication Type]) |
| #20 | (meta analysis[Title/Abstract]) OR (meta analyses[Title/Abstract]) OR (metaanalysis[Title/Abstract]) OR (metaanalyses[Title/Abstract]) OR (metanalysis[Title/Abstract]) OR (metanalyses[Title/Abstract]) OR (met-analysis[Title/Abstract]) OR (met-analyses[Title/Abstract]) OR (meta-study[Title/Abstract]) OR (meta-studies[Title/Abstract]) OR (meta study[Title/Abstract]) OR (meta studies[Title/Abstract]) OR (data pooling[Title/Abstract]) OR (data poolings[Title/Abstract]) OR (clinical trial overview[Title/Abstract]) OR (clinical trial overviews[Title/Abstract]) |
| #21 | "Systematic Reviews as Topic"[Mesh] OR (Systematic Review [Publication Type]) |
| #22 | (systematic review[Title/Abstract]) OR (systematic reviews[Title/Abstract]) OR (systematic study[Title/Abstract]) OR (systematic studies[Title/Abstract]) |
| #23 | #19 OR #20 OR #21 OR #22 |
| #24 | #11 AND #23 (Search systematic reviews for an overview of systematic reviews) |
| #25 | #11 AND #18 (Search primary studies for an update of systematic review) |

**Supplemental Table 2 Reason for excluded systematic reviews**

| References | Reason for Exclusion |
| --- | --- |
| 1. Kauark-Fontes E , Rodrigues-Oliveira L , Epstein J B , et al. Cost-effectiveness of photobiomodulation therapy for the prevention and management of cancer treatment toxicities: a systematic review[J]. Supportive Care in Cancer, 2021(8). | The outcome without regarding to the effectiveness and safety of PBMT |
| 2. Perazzini G , Trosini C , Materazzo M , et al. Physiotherapy for breast cancer patients: a critical review[J]. The Breast, 2019, 44:S135. | Conference abstract- Not SRs/MAs |
| 3. Mariana D P P , Araújo, Anna Luíza Damaceno, Arboleda, Lady Paola Aristizábal, et al. Tumor safety and side effects of photobiomodulation therapy used for prevention and management of cancer treatment toxicities. A systematic review[J]. Oral Oncology, 2019, 93:21-28. | Mixed participants with no (data for) subgroup analysis |
| 4. Jolien, Robijn, Sandrine, et al. The use of low-level light therapy in supportive care for patients with breast cancer: review of the literature[J]. Lasers in Medical Science, 2017, 32 (1):229-242. | Review- Not SRs/MAs |
| 5. Baxter GD, Liu L, Petrich S, et al. Low level laser therapy photobiomodulation for the management of breast cancer-related lymphoedema: An update[J]. Lasers in medical science. 2017, 32 (8):1704. | Conference abstract- Not SRs/MAs |
| 6. Li L , Yuan L , Chen X , et al. Current Treatments for Breast Cancer-Related Lymphoedema: A Systematic Review[J]. Asian Pacific Journal of Cancer Prevention Apjcp. 2016, 17(11):4875-4883. | Overview- Not SRs/MAs |
| 7. Baxter GD, Liu L, Chapple CM, et al.Photobiomodulation laser therapy for lymphoedema: Current evidence[J]. Lasers in medical science. 2015, 30 (8):2043. | Conference abstract |
| 8. Jeffs E , Bick D . The effectiveness of decongestive treatments provided within 12 months of developing a swollen arm for women with breast cancer-related lymphedema: a systematic review protocol of quantitative evidence[J]. Jbi Database of Systematic Reviews & Implementation Reports, 2014, 12(11):105. | Protocol- Not SRs/MAs |
| 9. Omar MTA, Shaheen AAM, Zafar H. A systematic review of the effect of low-level laser therapy in the management of breast cancer-related lymphedema[J]. Vasomed.2013, 25 (3):161-162 | Note- Not SRs/MAs |
| 10. Leal NF, Carrara HH, Vieira KF, et al. Physiotherapy treatments for breast cancer-related lymphedema: a literature review[J]. Revista latino-americana de enfermagem. 2009, 17 (5):730-736. | Review- Not SRs/MAs |

**Supplemental Table 3 Reason for excluded randomized controlled trials**

| References | Reason for Exclusion |
| --- | --- |
| 1. Turgay T, Denkeken T. The effect of low-level laser therapy on quality of life in postmastectomy lymphedema patients[J]. Biomedical Research and Therapy, 2020, 7(9):3971-3976. | Not RCT |
| 2. Photobiomodulation Therapy for the Management of Breast Cancer-related Lymphedema (2020). | Register of Controlled Trials |
| 3. Efficacy of low-level laser and complete decongestive therapy in the management of post-breast cancer related lymphedema (2017). | Register of Controlled Trials |
| 4. Laser Therapy for Lymphedema: feasibility Trial (2016). | Register of Controlled Trials |
| 5. LLLT Combined With CDT in Breast Cancer-Related Lymphedema (2011). | Register of Controlled Trials |
| 6. Dirican A , Andacoglu O , Johnson R , et al. The short-term effects of low-level laser therapy in the management of breast-cancer-related lymphedema.[J]. Supportive Care in Cancer, 2011, 19(5):685-690. | Not RCT |
| 7. Lymphedema and laser therapy in women with breast cancer (2010). | Register of Controlled Trials |
| 8. Low Level Laser Treatment and Breast Cancer Related Lymphedema (2009). | Register of Controlled Trials |
| 9. A randomised controlled trial of low level laser therapy for the treatment of lymphoedema secondary to breast cancer (2004). | Register of Controlled Trials |
| 10. Piller N B, Thelander A . Treatment of chronic postmastectomy lymphedema with low level laser therapy: a 2.5 year follow-up.[J]. Lymphology, 1998, 31(2):74-86. | Not RCT |

**Supplemental Table 4 The therapeutic effects of LLLT separately depending on the type of comparison (control)**

| **Comparison 1: LLLT versus Pneumatic compression** | | | | | |
| --- | --- | --- | --- | --- | --- |
| Outcome | Assessment time | Author | Year | Effect size | 95%CI |
| Limb circumference difference | post-treatment | Kozanoglu | 2009 | SMD 0.05 | (-0.52, 0.63) |
|  | 3-month follow up |  |  | SMD -0.20 | (-0.78, 0.37) |
|  | 6-month follow up |  |  | SMD -0.02 | (-0.59, 0.55) |
|  | 12-month follow up |  |  | **SMD -0.64** | **(-1.22, -0.05)** |
| Pain | post-treatment |  |  | SMD -0.10 | (-0.67, 0.48) |
|  | 3-month follow up |  |  | SMD -0.30 | (-0.88, 0.27) |
|  | 6-month follow up |  |  | **SMD -0.71** | **(-1.30, -0.12)** |
|  | 12-month follow up |  |  | **SMD -0.66** | **(-1.25, -0.07)** |
| Grip Strength | post-treatment |  |  | SMD -0.08 | (-0.65, 0.50) |
|  | 3-month follow up |  |  | SMD 0.04 | (-0.53, 0.61) |
|  | 6-month follow up |  |  | SMD -0.06 | (-0.63, 0.51) |
|  | 12-month follow up |  |  | SMD -0.17 | (-0.74, 0.40) |
| Tightness | 12-month follow up |  |  | OR 1.10 | (0.35, 3.48) |
| Heaviness | 12-month follow up |  |  | OR 0.65 | (0.21, 2.06) |
| Paraesthesia | 12-month follow up |  |  | OR 1.06 | (0.30, 3.71) |
| Weakness | 12-month follow up |  |  | OR 0.91 | (0.20, 2.49) |
| **Comparison 2: LLLT versus Compression bandage** | | | | | |
| Limb volume difference | post-treatment | Maiya | 2008 | SMD -1.21 | (-2.16, -0.25) |
| Pain | post-treatment |  |  | SMD -2.94 | (-4.21, -1.68) |
| **Comparison 3: LLLT versus MLD versus Combined LLLT and MLD** | | | | | |
| **LLLT versus MLD** | | | | | |
| Limb volume difference | post-treatment | Ridner | 2013 | SMD -0.19 | (-0.90, 0.52) |
| Exratcellular fluid | post-treatment |  |  | SMD -0.05 | (-0.76, 0.65) |
| Number of symptoms | post-treatment |  |  | SMD 0.12 | (-0.58, 0.83) |
| Overall symptom burden | post-treatment |  |  | SMD 0.06 | (-0.64, 0.77) |
| Fatigue | post-treatment |  |  | SMD 0.27 | (-0.44, 0.97) |
| Quality of life 1 | post-treatment |  |  | SMD -0.10 | (-0.80, 0.61) |
| Quality of life 2 | post-treatment |  |  | SMD 0.65 | (-0.07, 1.37) |
| **Combined LLLT and MLD versus MLD** | | | | | |
| Limb volume difference | post-treatment | Ridner | 2013 | SMD -0.12 | (-0.83, 0.58) |
| Exratcellular fluid | post-treatment |  |  | SMD -0.10 | (-0.80,0.61) |
| Number of symptoms | post-treatment |  |  | SMD 0.26 | (-0.45, 0.97) |
| Overall symptom burden | post-treatment |  |  | SMD -0.03 | (-0.73, 0.68) |
| Fatigue | post-treatment |  |  | SMD 0.38 | (-0.33, 1.09) |
| Quality of life 1 | post-treatment |  |  | SMD -0.48 | (-1.20, 0.23） |
| Quality of life 2 | post-treatment |  |  | SMD 0.71 | (-0.02, 1.44) |
| **Comparison 4: LLLT plus Conventional therapy versus Conventional therapy** | | | | | |
| Limb circumference difference | post-treatment | Baxter | 2018 | SMD 0.40 | (-0.59, 1.39) |
|  | 6-week follow up |  |  | SMD 0.04 | (-0.94, 1.02) |
| Pain | post-treatment |  |  | SMD -0.36 | (-1.35, 0.63) |
|  | 6-week follow up |  |  | SMD 0.31 | (-0.67, 1.30) |
| Heaviness | post-treatment |  |  | SMD -0.03 | (-1.01, 0.95) |
|  | 6-week follow up |  |  | SMD 0.42 | (-0.57, 1.41) |
| Self-consciousness | post-treatment |  |  | SMD -0.17 | (-1.15, 0.81) |
|  | 6-week follow up |  |  | SMD -0.52 | (-1.52, 0.48) |
| Anxiety | post-treatment |  |  | SMD 0.04 | (-0.94, 1.02) |
|  | 6-week follow up |  |  | SMD 0.00 | (-0.98, 0.98) |
| Perception of arm swelling | post-treatment |  |  | SMD -0.75 | (-1.77, 0.27) |
|  | 6-week follow up |  |  | SMD -0.32 | (-1.31, 0.67) |
| Emotion | post-treatment |  |  | SMD -0.77 | (-1.79, 0.25) |
|  | 6-week follow up |  |  | SMD -0.95 | (-1.99, 0.09) |
| **Comparison 5: Active LLLT plus CDT versus Inactive laser plus CDT** | | | | | |
| Limb volume difference | post-treatment (8 sessions) | Kilmartin | 2020 | OR 0.22 | (0.02, 2.67) |
|  | post-treatment (16 sessions) |  |  | OR 1.00 | (0.11, 9.23) |
|  | 3-month follow up |  |  | OR 4.64 | (0.16, 135.57) |
|  | 6-month follow up |  |  | OR 0.70 | (0.05, 10.01) |
|  | 12-month follow up |  |  | OR 0.14 | (0.01, 3.47) |
| Number of symptoms | post-treatment (8 sessions) |  |  | OR 3.32 | (0.12, 91.60) |
|  | post-treatment (16 sessions) |  |  | OR 0.67 | (0.08, 5.30) |
|  | 3-month follow up |  |  | OR 0.33 | (0.03, 4.19) |
|  | 6-month follow up |  |  | OR 0.20 | (0.02, 2.39) |
|  | 12-month follow up |  |  | OR 0.25 | (0.02, 3.10) |
| **Comparison 6: LLLT versus Placebo laser** | | | | | |
| Limb circumference difference | post-treatment | Omar | 2011 | **SMD 1.17** | **(0.59, 1.75)** |
|  | 1-month follow up |  |  | **SMD 0.88** | **(0.32, 1.44)** |
| Grip Strength | post-treatment |  |  | SMD 0.51 | (-0.03, 1.05) |
|  | 1-month follow up |  |  | **SMD 0.95** | **(0.39, 1.95)** |
| Shoulder ROM (Flexion) | post-treatment |  |  | **SMD 1.11** | **(0.53, 1.68)** |
| Shoulder ROM (Abduction) | post-treatment |  |  | **SMD 1.75** | **(1.12, 2.38)** |
| Shoulder ROM (External rotation) | post-treatment |  |  | SMD 0.16 | (-0.37, 0.70) |
| Limb volume difference | post-treatment | Stora | 2017 | SMD 0.27 | (-0.39, 0.93) |
| Pain | post-treatment |  |  | SMD 0.00 | (-0.65, 0.65) |
|  | 1-month follow up |  |  | SMD 0.00 | (-0.65, 0.65) |
|  | 2-month follow up |  |  | SMD 0.00 | (-0.65, 0.65) |
|  | 3-month follow up |  |  | SMD 0.60 | (-0.07, 1.27) |
| Grip Strength | post-treatment |  |  | SMD -0.29 | (-0.94, 0.37) |
|  | 1-month follow up |  |  | SMD -0.37 | (-1.03, 0.29) |
|  | 2-month follow up |  |  | SMD 0.11 | (-0.54, 0.77) |
|  | 3-month follow up |  |  | SMD -0.24 | (-0.90, 0.41) |
| Quality of life (MMSQ) | post-treatment |  |  | SMD 0.17 | (-0.49, 0.82) |
|  | 1-month follow up |  |  | SMD -0.31 | (0.97, 0.35) |
|  | 2-month follow up |  |  | SMD 0.13 | (-0.52, 0.79) |
|  | 3-month follow up |  |  | SMD 0.10 | (-0.56, 0.75) |
| Quality of life (MQOL) | post-treatment |  |  | SMD 0.65 | (-0.02, 1.32) |
|  | 1-month follow up |  |  | SMD 0.06 | (-0.60, 0.71) |
|  | 2-month follow up |  |  | SMD 0.38 | (-0.28, 1.04) |
|  | 3-month follow up |  |  | SMD 0.10 | (-0.56, 0.75) |
| Activity disability (1 cycle of LLLT) | post-treatment | Carati | 2003 | SMD -0.41 | (-0.91, 0.10) |
|  | 1-month follow up |  |  | **SMD 1.52** | **(0.95, 2.09)** |
|  | 2-3-month follow up |  |  | **SMD 1.14** | **(0.60, 1.68)** |
| Quality of life (1 cycle of LLLT) | post-treatment |  |  | **SMD -0.67** | **(-1.19, -0.15)** |
|  | 1-month follow up |  |  | SMD 0.00 | (-0.50, 0.50) |
|  | 2-3-month follow up |  |  | **SMD -0.67** | **(-1.19, -0.15)** |
| Activity disability (2 cycles of LLLT) | post-treatment |  |  | SMD -0.33 | (-0.88, 0.21) |
|  | 1-month follow up |  |  | SMD 0.00 | (-0.54, 0.54) |
|  | 2-3-month follow up |  |  | SMD 0.32 | (-0.23, 0.86) |
| Quality of life (2 cycles of LLLT) | post-treatment |  |  | **SMD -1.26** | **(-1.86, -0.67)** |
|  | 1-month follow up |  |  | **SMD -0.63** | **(-1.19, -0.07)** |
|  | 2-3-month follow up |  |  | **SMD -1.90** | **(-2.56, -1.24)** |
| Limb volume difference (2 cycles of LLLT) | post-treatment |  |  | SMD 0.09 | (-0.46, 0.63) |
|  | 1-month follow up |  |  | SMD -0.35 | (-0.90, 0.20) |
| **Comparison 7: LLLT versus No treatment** | | | | | |
| Limb volume difference | post-treatment | Lau | 2009 | SMD -0.53 | (-1.41, 0.34) |
|  | 1-month follow up |  |  | **SMD -1.01** | **(-1.92, -0.09)** |
| Tissue resistance (site 1) | post-treatment |  |  | SMD 0.69 | (-0.20, 1,57) |
|  | 1-month follow up |  |  | **SMD 1.30** | **(0.35, 2.25)** |
| Tissue resistance (site 2) | post-treatment |  |  | SMD 0.48 | (-0.39, 1.35) |
|  | 1-month follow up |  |  | SMD 0.71 | (-0.17, 1.60) |
| Tissue resistance (site 3) | post-treatment |  |  | SMD -0.17 | (-1.03, 0.69) |
|  | 1-month follow up |  |  | SMD 0.33 | (-0.53, 1.20) |
| Tissue resistance (site 4) | post-treatment |  |  | SMD 0.62 | (-0.26, 1.50) |
|  | 1-month follow up |  |  | **SMD 0.95** | **(0.04, 1.85)** |
| Disabilities of Arm, Shoulder, and Hand (DASH) questionnaire | post-treatment |  |  | SMD -0.44 | (-1.31, 0.42) |
|  | 1-month follow up |  |  | SMD -0.71 | (-1.60, 0.18) |

LLLT, low-level laser therapy; SMD, standardized mean difference; OR, odd ratio; CI, confidence intervals; MLD, manual lymphatic drainage; CDT, complex decongestive therapy; ROM, range of motion; MMSQ, Multidimensional Mood State Questionnaire; MQOL, McGill Quality of Life Questionnaire.

**Supplemental Figure 1 Risk of bias summary for updated systematic review** **studies**


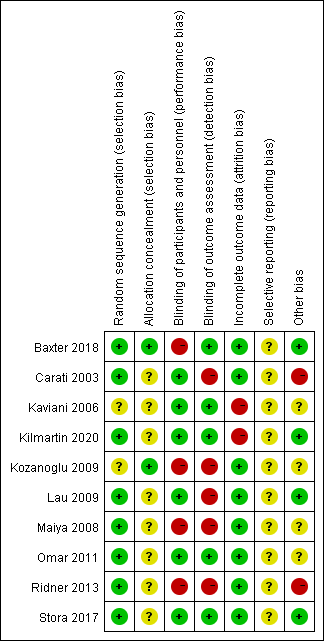


**Supplemental Figure 2 Risk of bias graph for updated systematic review studies**


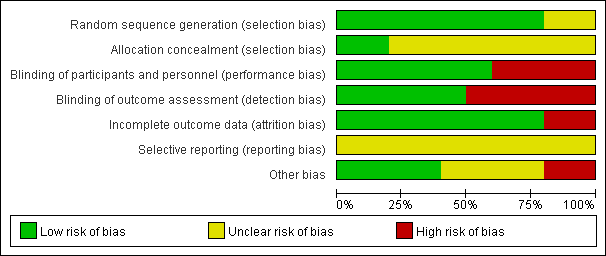

Supplement: Supplementary file 1 — Supplementary file1 (DOCX 58 KB) [file 10103_2021_3446_MOESM1_ESM.docx]
